# Effectiveness of oral semaglutide versus empagliflozin for the management of type 2 diabetes. PIONEER‐2 trial emulation with real‐world data

**DOI:** 10.1111/dom.70151

**Published:** 2025-09-24

**Authors:** Gian Paolo Fadini, Enrico Longato, Sara Poletto, Andrea Giaccari, Mariangela Ghiani, Marco Strazzabosco, Maddalena Trombetta, Giuseppe Penno, Angelo Avogaro, Anna Solini, Agostino Consoli, GLIMPLES study investigators and DARWIN‐Renal study investigators, Maria Antonia Pompea Baldassarre, Gloria Formoso, Agostino Consoli, Gaetano Leto, Frida Leonetti, Stefano Fazion, Giancarla Meregalli, Marco Zavattaro, Gianluca Aimaretti, Elena Melchionda, Cristina Barale, Rosella Cau, Mariangela Ghiani, Andrea Muscarà, Giuseppina Russo, Roberto Anichini, Bruno Fattor, Gian Paolo Fadini, Angelo Avogaro, Laura Nollino, Agostino Paccagnella, Marco Strazzabosco, Mariella Baldassarre, Agostino Consoli, Sara Morganet, Antonella Zugaro, Marco Giorgio Baroni, Francesco Andreozzi, Adriano Gatti, Stefano De Riu, Andrea Del Buono, Raffaella Aldigeri, Riccardo Bonadonna, Alessandra Dei Cas, Angela Vazzana, Monica Antonini, Valentina Moretti, Patrizia Li Volsi, Miranda Cesare, Giorgio Zanette, Silvia Carletti, Paola D'Angelo, Gaetano Leto, Frida Leonetti, Luca D'Onofrio, Ernesto Maddaloni, Raffaella Buzzetti, Fabiana Picconi, Simona Frontoni, Gisella Cavallo, Susanna Morano, Tiziana Filardi, Francesca Cinti, Andrea Giaccari, Antonio C. Bossi, Giancarla Meregalli, Fabrizio Querci, Alessia Gaglio, Veronica Resi, Emanuela Orsi, Stefano Fazion, Ivano G. Franzetti, Cesare Berra, Silvia Manfrini, Gabriella Garrapa, Giulio Lucarelli, Lara Riccialdelli, Elena Tortato, Marco Zavattaro, Gianluca Aimaretti, Franco Cavalot, Guglielmo Beccuti, Fabio Broglio, Bruno Fattor, Giuliana Cazzetta, Olga Lamacchia, Anna Rauseo, Salvatore De Cosmo, Rosella Cau, Mariangela Ghiani, Antonino Di Benedetto, Antonino Di Pino, Salvatore Piro, Francesco Purrello, Lucia Frittitta, Agostino Milluzzo, Giuseppina Russo, Anna Solini, Monia Garofolo, Giuseppe Penno, Stefano Del Prato, Roberto Anichini, Gian Paolo Fadini, Angelo Avogaro, Lucia Gottardo, Mauro Rigato, Agostino Paccagnella, Marco Strazzabosco, Massimo Cigolini, Enzo Bonora

**Affiliations:** ^1^ Department of Medicine University of Padova Padua Italy; ^2^ Laboratory of Experimental Diabetology, Veneto Institute of Molecular Medicine Padua Italy; ^3^ Department of Information Engineering University of Padova Padua Italy; ^4^ Endocrine and Metabolic Center Fondazione Policlinico Universitario A. Gemelli IRCCS and Università Cattolica del Sacro Cuore Rome Italy; ^5^ Diabetology Unit, Azienda Sanitaria Locale 8 Cagliari Quartu S. Elena Cagliari Italy; ^6^ Diabetology and Metabolic Diseases Unit, S. Bortolo Hospital Vicenza Italy; ^7^ Endocrinology, Diabetology and Metabolic Diseases University of Verona and University Hospital of Verona Verona Italy; ^8^ Department of Clinical and Experimental Medicine University of Pisa Pisa Italy; ^9^ Department of Surgical, Medical, Molecular and Critical Area Pathology University of Pisa Pisa Italy; ^10^ Endocrinology and Metabolism Unit ASL Pescara Italy; ^11^ Department of Medicine and Aging Sciences DMSI and Center for Advanced Studies and Technology CAST “G. D'Annunzio” University of Chieti‐Pescara Chieti Italy

**Keywords:** cohort study, empagliflozin, real‐world evidence, semaglutide

## Abstract

**Background and Aims:**

Oral semaglutide and empagliflozin are commonly used for the management of type 2 diabetes (T2D), but head‐to‐head comparisons in real‐world settings are limited. We aimed to emulate the PIONEER‐2 trial using electronic health records to compare the effectiveness and persistence of oral semaglutide versus empagliflozin.

**Methods:**

This was a retrospective multicentre study using electronic health records from Italian diabetes clinics. New users of oral semaglutide or empagliflozin were matched 1:2 and followed for up to 18 months. The primary outcome was HbA1c change; secondary outcomes included weight change and treatment persistence. Analyses used mixed models for repeated measures under both treatment policy and trial product estimands.

**Results:**

After matching, we included new users of oral semaglutide (*n* = 105) or empagliflozin (*n* = 207). Mean age was 65 years, diabetes duration 10 years, baseline HbA1c 7.6%, BMI 29 kg/m^2^, and 94% were on metformin. Only 28.6% of new users of oral semaglutide reached the 14 mg dose and 31.4% of empagliflozin new users reached the 25 mg dose. HbA1c reduction was significantly greater with oral semaglutide than with empagliflozin (mean difference − 0.35%, *p* <0.001). Weight loss over time was similar, with oral semaglutide showing a modest advantage at 18 months among persistent patients. Persistence was lower for semaglutide (HR for discontinuation 1.47, *p* = 0.007).

**Conclusions:**

Under routine care, new users of oral semaglutide achieved better glycaemic control compared with empagliflozin new users, with similar weight loss but lower treatment persistence. These findings support the results of PIONEER‐2 and its transferability to clinical practice.

## 1 INTRODUCTION

Type 2 diabetes (T2D) is a chronic progressive disorder imposing a tremendous global health burden,^1^ mainly due to its complications.^2^, ^3^ It is estimated to affect 7.5% of the Italian population.^4^ The clinical management of T2D emphasises comprehensive strategies that incorporate stringent glucose control, targeting blood pressure and lipids, achieving healthy body weight, and delaying or preventing microvascular and macrovascular complications.^5^

Within treatment algorithms, glucagon‐like peptide‐1 receptor agonists (GLP‐1RA) and sodium–glucose cotransporter‐2 inhibitors (SGLT2i) are foundational options, especially for patients with established atherosclerotic cardiovascular disease, heart failure, or chronic kidney disease.^6^ The 2023 update of the Italian guidelines for the treatment of T2D positions SGLT2i as first‐line treatment in those with CKD or heart failure, and GLP‐1RA or SGLT2i as first‐line treatment in those with previous cardiovascular events.^7^

These two classes of glucose‐lowering agents have different modes of action and complementary effects. GLP‐1RAs act by augmenting glucose‐dependent insulin secretion, suppressing glucagon, and promoting weight loss,^8^ while SGLT2is improve glycaemic control via renal glucose excretion and confer cardiorenal protection through natriuresis and haemodynamic effects.^9^ Network meta‐analyses consistently show that GLP‐1RAs allow greater reductions in HbA1c, while the difference in body weight change depends on the type of GLP‐1RA being investigated.^10^ The advent of oral semaglutide, the first orally administered GLP‐1RA, has fostered the interest in comparing it with other oral diabetes drugs, such as SGLT2i. This was addressed by the PIONEER‐2 study, a 52‐week, randomised trial involving oral semaglutide (14 mg) versus empagliflozin (25 mg) add‐on to metformin.^11^ At week 26, oral semaglutide achieved significantly greater HbA1c reduction compared to empagliflozin (−1.3% vs. −0.9%; estimated treatment difference −0.4%; *p* <0.0001), but weight changes were similar. By week 52, semaglutide demonstrated superiority in both HbA1c and weight reduction (mean weight loss −4.7 kg vs. −3.8 kg; *p* = 0.0114), though gastrointestinal adverse events were more frequent.

In the prospective PIONEER REAL Italy study, oral semaglutide (*n* = 398) reduced HbA1c by 0.9% (from a baseline of 7.8%) and body weight by 3.8 kg (from a baseline of 87.8 kg).^12^ These results were consistent with those obtained in the PIONEER REAL programme in other countries^13^ and in other real‐world studies.^14^

Building on PIONEER‐2, the present study aims to emulate its design using real‐world electronic health record data. Our objectives were to compare the effectiveness of oral semaglutide versus empagliflozin on HbA1c and weight trajectories, as well as evaluate treatment persistence. By doing so, we seek to bridge the gap between randomised trial efficacy and routine clinical effectiveness, providing insights to inform treatment strategies in T2D management in everyday practice.

## 2 METHODS

### 2.1 Study design

This was a retrospective multicentre real‐world study promoted by the Italian Diabetes Society and conducted in specialised diabetes care centres in Italy. Data were extracted from the same electronic chart system at all centres and were anonymised at the time of extraction as recommended by approved standards.^15^ The study was conducted in accordance with the Declaration of Helsinki and approved by the local ethical committee at all participating centres. In agreement with National Regulations on retrospective studies on anonymised data, the need for patients' informed consent was waived.

### 2.2 Cohorts and exposures

We included in this study patients regularly followed at the participating outpatient clinics. Inclusion criteria were: age 18–80 years; a diagnosis of T2D as reported in the electronic record; baseline HbA1c >6.5% (48 mmol/mol); initiation of oral semaglutide or empagliflozin up to December 2021 (closure of the retrospective collection of drug initiation events); availability of at least one HbA1c value post‐index date for evaluation of the primary outcome. Exclusion criteria were: other forms of diabetes; age < 18 or >80 years; missing data for the primary outcome. Participants were divided into two groups: (i) new users of oral semaglutide; (ii) new users of empagliflozin. New use was defined as a first prescription of the index drugs in patients who had not been treated with any GLP‐1RA or SGLT2i in their entire past history, as recorded in the chart. The index date was defined as the date of the first prescription of the index drug. As recommended for clinical trials,^16^ we defined two estimands. For the treatment policy estimand, participants were considered to be exposed to the index drug until the last available observation, whether or not the drug continued to be prescribed. This informs on effectiveness in real‐world practice, as this estimand reflects what happens when a treatment is initiated and then managed according to routine care. For the trial product estimand, patients were considered to be exposed as long as the index drug continued to be prescribed and were therefore censored at the time of drug discontinuation. Clinically, this estimand isolates the pharmacological effect of the drug itself, independent of persistence.

There was no information on pharmacy refill rates and adherence. Persistence was defined as the confirmation of the prescription at follow‐up visits, whereas non‐persistence was defined when the index drug was no longer prescribed at a follow‐up visit. As each visit reported the list of medications that the specialist prescribed, the date of discontinuation was set as the date when the drug was no longer included in the outpatient medication list.

### 2.3 Definition of variables

We collected the following information at baseline, as recorded in the electronic chart. Consistent with the retrospective real‐world nature of the study, methods for recording variables were not standardised. Demographics: age, sex, duration of diabetes. Anthropometrics: body weight and height to calculate the BMI in kg/m^2^. Vital parameters: systolic and diastolic blood pressure measured during routine visits. Laboratory values: fasting glucose, HbA1c, lipid profile (LDL was calculated using the Friedewald formula^17^), urinary albumin/creatinine ratio (UACR, normalised as described in Reference [^18^]), and serum creatinine to calculate the estimated glomerular filtration rate (eGFR) with the CKD‐EPI equation.^19^ Complications (retinopathy was defined based on digital retinal examination; cardiovascular disease was defined as a history of stroke, myocardial infarction, or arterial revascularization); medications for the treatment of diabetes and concomitant cardiovascular risk factors. No information was available on adverse events.

At each subsequent visit available in the chart, we recorded updated values of body weight, HbA1c, serum lipids, eGFR, and UACR, along with the prescribed therapy.

### 2.4 Outcomes

The primary endpoint was the change in HbA1c from baseline through follow‐up visits until 18 months. Secondary endpoints included: the proportion of participants reaching HbA1c ≤6.5%, the change in body weight, the proportion of participants reaching a weight loss of 5% or more, the change in fasting serum triglycerides, eGFR and UACR.

### 2.5 Statistical analysis

Continuous variables are presented as mean (SD), while categorical variables are presented as number (percentages). Normality of data distribution was assessed using the Shapiro–Wilk test, and non‐normal variables were log‐transformed before analysis with parametric tests. The comparison of continuous variables was performed with the Student's *t* test, whereas categorical variables were compared with the chi‐square test. To handle missing values, we performed a single multivariable imputation by chained equation (mice package, version 3.16.0), employing default settings for both predictor matrix and where, with all variables used as predictors. Propensity score matching (PSM) was then performed on the imputed dataset using the MatchIt package (version 4.5.5). Propensity scores were estimated through logistic regression, including all covariates, and matching was conducted using nearest‐neighbour matching with a 1:2 ratio (treated: control), without replacement. The optimal calliper width, determined by iteratively testing values from 0.1 to 1 in increments of 0.05, was set at 0.15 times the standard deviation of the estimated propensity scores. Control subjects outside the region of common support were excluded, and the propensity score model was refit on the restricted sample. Balance diagnostics to demonstrate satisfactory covariate balance post‐matching were based on absolute standardised mean differences (SMD) below 0.1. The 1:2 ratio was chosen because of the large imbalance in initial sample sizes, to enable efficient use of available data, improved covariate balance without overfitting, and minimising residual confounding. The change over time in outcome measures was analysed with the mixed model for repeated measures (MMRM). The outcome variable (e.g., HbA1c) was the dependent variable, fixed factors included time, group (exposure to oral semaglutide or empagliflozin), time‐by‐group interaction, and baseline outcome variable. As variance structure, the first‐order autoregressive was chosen. In sensitivity analyses, drug dosage was entered as a covariate of the MMRM, and the adjusted effect size is reported. Persistence was analysed as a time‐to‐event endpoint using the Cox proportional hazard model. The conventional threshold of *p* <0.05 was chosen to define statistical significance, without hierarchical testing of the various endpoints. All analyses were conducted in SPSS version 21 or later and in R version 4.4.1.

## 3 RESULTS

### 3.1 Patients

The databases contained initial information on *n* = 166 new users of oral semaglutide and *n* = 6643 new users of empagliflozin (Figure S1). After applying inclusion/exclusion criteria, *n* = 109 and *n* = 3757 participants remained in each group. Before matching, the two groups were imbalanced for several key clinical characteristics. New users of oral semaglutide were older, with a longer disease duration, lower BMI, a different profile of blood pressure and lipids, a greater prevalence of CKD but a lower prevalence of cardiovascular disease, and a different combination of concomitant medications (Table S1 and Figure S2).

After matching, *n* = 105 new users of oral semaglutide and *n* = 207 new users of empagliflozin were included. All variables shown in Table 1 were well balanced (SMD <0.1). Participants (64% men) were on average 65 years old, with a diabetes duration of about 10 years, BMI of 29 kg/m^2^, baseline HbA1c of 7.6%. The prevalence of CKD stage III or greater was 17% and that of established cardiovascular disease was 12%. The vast majority of patients were on metformin, with small percentages also taking sulphonylurea, pioglitazone, or basal insulin (<7%).

**TABLE 1:** Characteristics of study participants.

	Oral semaglutide	Empagliflozin	SMD
Demographics	*n* = 105	*n* = 207	
Male sex, %	68 (64.8)	131 (63.3)	0.03
Age, years	64.6 (8.7)	64.8 (8.0)	0.03
Duration, years	10.1 (7.9)	9.9 (8.4)	0.01
Risk factors and lab results			
Body weight, kg	82.9 (17.9)	82.2 (16.9)	0.05
Body mass index, kg/m^2^	29.1 (5.5)	29.2 (4.9)	0.01
Systolic blood pressure, mm Hg	143.3 (19.3)	142.3 (21.0)	0.04
Diastolic blood pressure, mm Hg	80.2 (10.0)	80.3 (10.2)	0.01
Fasting plasma glucose, mg/dL	158.1 (49.2)	160.0 (44.4)	0.03
HbA1c, %	7.6 (1.1)	7.7 (0.9)	0.05
Total cholesterol, mg/dL	167.7 (35.9)	168.4 (38.2)	0.04
HDL cholesterol, mg/dL	49.6 (13.2)	49.3 (14.7)	0.03
LDL cholesterol, mg/dL	88.4 (32.0)	89.9 (31.9)	0.07
Triglycerides, mg/dL	125.8 (85.5; 185.2)	129.3 (91.3; 183.1)	0.00
eGFR, mL/min/1.73 m^2^	83.7 (18.9)	83.0 (17.0)	0.05
Complications			
eGFR <60 mL/min/1.73 m^2^	17 (16.2)	38 (18.4)	0.08
UACR >30 mg/g	24 (22.9)	41 (19.8)	0.07
Retinopathy, %	17 (16.2)	30 (14.5)	0.03
Cardiovascular disease, %	13 (12.4)	23 (11.1)	0.04
Medications			
Metformin, %	98 (93.3)	196 (94.7)	0.06
Sulphonylurea, %	11 (10.5)	20 (9.7)	0.02
Pioglitazone, %	4 (3.8)	7 (3.4)	0.00
Basal insulin, %	7 (6.7)	14 (6.8)	0.00
Statin, %	76 (72.4)	156 (75.4)	0.05
Anti‐platelet agents, %	35 (33.3)	65 (31.4)	0.04
RAS blockers, %	65 (61.9)	121 (58.5)	0.07
Beta‐blockers, %	28 (26.7)	60 (29.0)	0.04
Calcium channel blockers, %	21 (20.0)	43 (20.8)	0.01
Diuretics, %	20 (19.0)	40 (19.3)	0.00

*Note*: Data are presented as mean (standard deviation) for continuous variables, except for triglycerides, expressed as median (interquartile range), or as number (percentage) for categorical variables. The standardised mean difference (SMD) is shown for the between‐group comparison after matching. There was no significant between‐group difference in any variable (all *p* >0.05).

Abbreviations: eGFR, estimated glomerular filtration rate; RAS, renin angiotensin system; UACR, urinary albumin/creatinine ratio.

### 3.2 Exposure and persistence

Oral semaglutide was initiated at a dose of 3 mg, and the maximal dose was 3 mg in 27.6% of patients, 7 mg in 43.8% of patients, and 14 mg in 28.6% of patients. Empagliflozin was started at 10 mg in 76.6% of patients and at 25 mg in 23.4%. The final dose was 10 mg in 68.6% and 25 mg in the remaining 31.4%. The observation was closed at 18 months because the number of patients in the oral semaglutide group dropped substantially after that time point.

The rate of discontinuation was significantly higher in the oral semaglutide group than in the empagliflozin group, with an HR of 1.47 (95% C.I. 1.11–1.91; *p* = 0.007; Figure S3).

### 3.3 Glycaemic control

**FIGURE 1:**
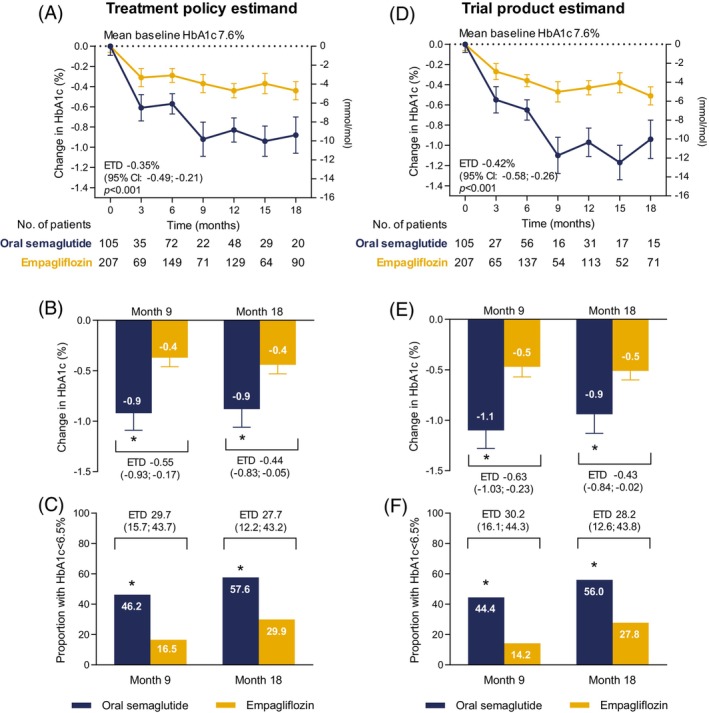
Glycaemic efficacy of oral semaglutide versus empagliflozin. Results are presented for the treatment policy estimand (A)–(C) and for the trial product estimand (D)–(F). (A) and (D) Estimated absolute change in HbA1c over time. (B) and (E) Estimated changes from baseline in HbA1c at months 9 and 18. (C) and (F) Estimated proportions of patients achieving HbA1c <6.5% (48 mmol/mol) at months 9 and 18. CI, confidence interval; ETD, estimated treatment difference.

The analysis was based on a median (IQR) of 4 (2–6) values per patient, with a greater frequency for empagliflozin (median 5; IQR 2–8) than for oral semaglutide (median 3; IQR 2–4; *p* <0.001). HbA1c improved significantly more in the oral semaglutide group than in the empagliflozin group (Figure 1). According to the treatment policy estimand, from a baseline of 7.6% in both groups, the mean difference in HbA1c was −0.35% (95% C.I. from −0.49 to −0.21; *p* <0.001; Figure 1A) in favour of oral semaglutide. The estimated treatment difference was −0.55% at 9 months and −0.44% at 18 months (Figure 1B). The effect size remained similar after adjusting for drug dosages (mean HbA1c difference −0.38%; *p* <0.001). The trial product estimand yielded slightly greater differences between the two groups, with a mean HbA1c difference of −0.42% in favour of the oral semaglutide group (reaching −0.63% at 9 months and −0.43 at 18 months; Figure 1D,E). More participants in the oral semaglutide group achieved an HbA1c value ≤6.5%, which exceeded 50% at 18 months in the oral semaglutide group (Figure 1C,F).

### 3.4 Body weight

**FIGURE 2:**
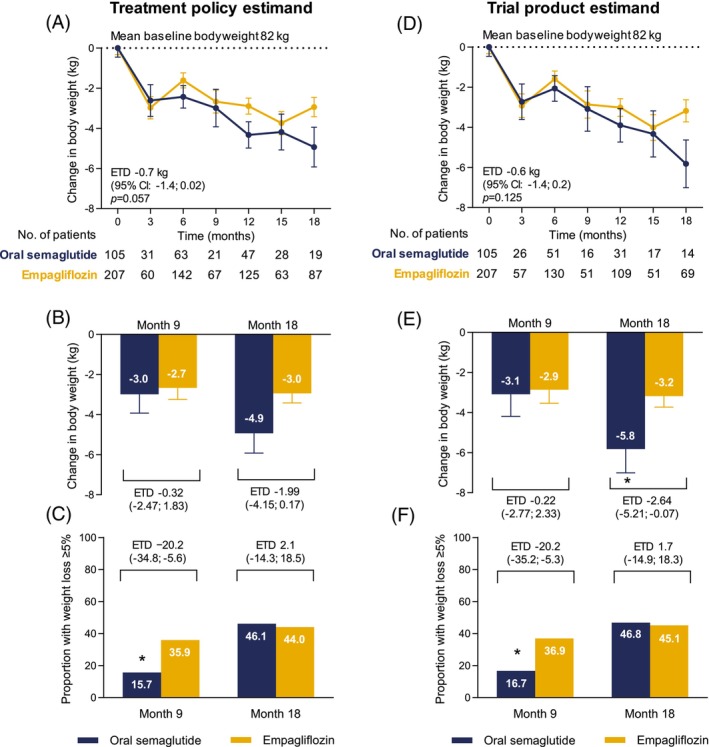
Body weight change and targets with oral semaglutide versus empagliflozin. Results are presented for the treatment policy estimand (A)–(C) and for the trial product estimand (D)–(F). (A) and (D) Estimated absolute change in body weight over time. (B) and (E) Estimated changes from baseline in body weight at months 9 and 18. (C) and (F) Estimated proportions of patients achieving weight reduction of 5% or more at months 9 and 18. CI, confidence interval; ETD, estimated treatment difference.

The change over time in body weight was similar between groups. For the treatment policy estimand and the trial product estimand, the mean difference was −0.7 kg (*p* = 0.057) and −0.6 kg (*p* = 0.125), respectively (Figure 2A,D). The difference became statistically significant after adjusting for drug dosages (mean difference −0.8 kg; *p* = 0.032). The estimated mean difference was very similar between groups at 9 months (about −3 kg; Figure 2B,E), while weight loss tended to be more pronounced in the oral semaglutide group at 18 months, which was nominally significant for the trial product estimand (−2.6 kg; 95% C.I. from −5.2 to −0.1; Figure 2E). At 9 months, significantly more participants in the empagliflozin group had lost 5% or more body weight, while the proportion was numerically in favour of oral semaglutide at 18 months (Figure 2C,F).

### 3.5 Other endpoints

The change over time in serum triglycerides, eGFR and UACR is presented in Table S2. No significant between‐group difference was observed for any of these additional endpoints according to the treatment policy estimand and the trial product estimand.

## 4 DISCUSSION

In this real‐world study emulating the PIONEER‐2 trial, oral semaglutide allowed a significantly greater reduction in HbA1c over 18 months compared to empagliflozin, while weight loss was overall comparable between groups, with a modest advantage for oral semaglutide at 18 months. Treatment persistence was lower with oral semaglutide, suggesting potential tolerability or adherence limitations in real‐life settings.

Our findings align with the PIONEER‐2 trial, but the study diverges in noteworthy key aspects (Table 2). In terms of design, PIONEER‐2 was a 52‐week, multicentre, open‐label trial with 822 participants randomised to oral semaglutide 14 mg or empagliflozin 25 mg, whereas the current study is a retrospective observational emulation using matched real‐world cohorts from Italian diabetes centres, including 105 patients on semaglutide and 207 on empagliflozin. The randomised nature of PIONEER‐2 grants a greater level of evidence and minimises bias, whereas the current study attempts to mitigate selection bias through matching, but residual confounding cannot be excluded. Participants in the real‐world study were older, had lower HbA1c and BMI, and a higher prevalence of CKD compared to those in PIONEER‐2, who were recruited and followed under strict trial procedures. The enrolment criteria were also different, as we included new users of oral semaglutide or empagliflozin with HbA1c >6.5%, while PIONEER‐2 participants had HbA1c between 7.0% and 10.5%. This lower limit of HbA1c was more permissive in our study, in line with the possibility to achieve more ambitious glycaemic targets with modern diabetes pharmacotherapy.^5^ The sample size in the real‐world study is smaller and, though follow‐up was extended to 18 months, a fraction of participants reached the 18 months timepoint, while PIONEER‐2 maintained a controlled 52‐week duration. Importantly, concomitant medications were also different: real‐world patients had varied background therapies reflecting clinical practice, whereas PIONEER‐2 restricted background treatment to metformin only.

**TABLE 2:** Comparison between the present real‐world study and the PIONEER‐2 trial.

	PIONEER‐2 trial	This real‐world study
Study characteristics		
Design	Randomised, open‐label, multinational trial	Retrospective multicenter observational study
Drug dosages	Oral semaglutide 14 mg Empagliflozin 25 mg	Oral semaglutide mostly 7 mg, empagliflozin mostly 10 mg
Eligibility HbA1c	7.0%–10.5%	>6.5%
Primary endpoint	Change in HbA1c from baseline to week 26	Change in HbA1c up to 18 months
Duration, weeks	52	78
Number of participants	821	312
Patient characteristics		
Age, years	57	64
Sex male, %	50.5	64
Diabetes duration, years	7.4	10
BMI, kg/m^2^	32.8	29
HbA1c, %	8.1	7.6
eGFR, ml/min/1.73 m^2^	95	83
Concomitant metformin, %	100	94
Main results under treatment policy estimand		
Change in HbA1c	−0.4%	−0.4%
Change in body weight	Non‐significant at 26 weeks (−2.1 kg at 52 weeks)	Non‐significant at 9 months (−2.0 kg at 18 months)
Discontinuation	Discontinuation due to adverse events was greater with oral semaglutide	Discontinuation was greater with oral semaglutide

Despite these notable differences, the direction of results was extremely consistent. We were able to confirm the superior glycaemic control with oral semaglutide compared to empagliflozin. The HbA1c difference in favour of oral semaglutide in our real‐world study (−0.4%) was closely aligned with that reported in PIONEER‐2 (from −0.4% to −0.5% depending on estimand), supporting the robustness of the finding across contexts. In the real world, a significant proportion of patients received submaximal doses of oral semaglutide (only 28.6% reached 14 mg), which is aligned with several reports on the use of oral semaglutide in many countries, including Italy, and showing that a minority of patients reached the 14 mg dose.^12^, ^13^, ^14^, ^20^ This may be related to the higher discontinuation rate with semaglutide, possibly reflecting tolerability issues that limit titration. In the empagliflozin group, most participants remained on the 10 mg dose, reflecting the marginal incremental effect of the 25 mg over the 10 mg dose in phase III trials and in cardiovascular outcome trials with empagliflozin.^21^, ^22^ The lower baseline HbA1c (7.6%) than in the PIONEER‐2 trial (8.1%), along with the lower drug doses, may have contributed to a lower HbA1c reduction with both oral semaglutide and empagliflozin in the real world compared to what was observed in the trial. Yet, the lower HbA1c at the time of drug initiation enabled the achievement of HbA1c values <6.5% in a considerable proportion of participants (nearly 60% among those who persisted on oral semaglutide at 18 months).

In addition to glycaemic control, weight management has become a major objective of comprehensive person‐centred T2D care.^23^ The PIONEER‐2 trial showed superiority of oral semaglutide for body weight loss at 52 weeks, but not at 26 weeks. Our real‐world study shows similar weight trajectories in the two groups. Weight outcomes with oral semaglutide may be more variable in clinical practice, potentially due to dose titration limitations. In this context, the lower persistence on oral semaglutide is a critical finding that may reflect gastrointestinal tolerability issues that were also prominent in PIONEER‐2. The lower real‐world doses of oral semaglutide may explain delayed weight loss observed in this study because weight reduction with GLP‐1RA is strongly dose‐dependent.^10^

To our knowledge, this is the first trial emulation directly comparing oral semaglutide with empagliflozin. In the PAUSE study, employing a propensity score‐based approach to reduce bias, new users of once weekly semaglutide had significantly greater improvements in body weight and HbA1c at 1 year than new users of SGLT2i.^24^ In a long‐term observation of >11 000 new users of GLP‐1RA (mainly dulaglutide) or SGLT2i (mainly dapagliflozin), we found that GLP‐1RA allowed a better and persistent glycaemic control, with a mean HbA1c difference of about 0.2%, but a less prominent weight loss.^25^ This was confirmed by another real‐world study^26^ and aligns with a network meta‐analysis comparing SGLT2i and GLP‐1RA, excluding high‐dose semaglutide/dulaglutide and tirzepatide.^10^ In terms of multiple endpoints, however, real‐world data suggest that therapy with GLP‐1RA and SGLT2i may be equivalent in achieving simultaneous benefits on HbA1c, body weight, and blood pressure.^27^ The heterogeneous results obtained across studies comparing GLP‐1RA and SGLT2i not only derive from the different designs and molecules being investigated but may rely on patient phenotypes, as illustrated by the MASTERMIND initiative,^28^ suggesting the possibility to individualise treatment based on patient characteristics.

For a clinical interpretation of our results, it is important to consider that comprehensive T2D management goes beyond HbA1c and body weight goals. Dedicated trials have demonstrated that oral semaglutide and empagliflozin exert notable protection against chronic diabetic complications. In the EMPA‐REG Outcome trial, in people with T2D and cardiovascular disease, empagliflozin reduced cardiovascular events, cardiovascular and all‐cause mortality, hospitalisation for heart failure and adverse kidney outcomes.^22^ Consistent results have been gathered from other trials confirming the strong protective effects of empagliflozin against heart failure and kidney outcomes.^29^ In the SOUL trial, among people with T2D and cardiovascular disease, oral semaglutide reduced the rates of cardiovascular events, with a prominent effect on myocardial infarction and peripheral atherosclerosis.^30^ In real life, we have previously demonstrated better cardiovascular outcomes for patients with T2D who received SGLT2i versus GLP‐1RA.^31^ Therefore, in agreement with local and international guidelines, therapeutic personalisation should consider these demonstrated benefits on hard outcomes in addition to the probability of achieving glycaemic and weight targets.

We wish to acknowledge important study limitations. The observational non‐randomised design is intrinsically prone to bias, mainly due to unmeasured confounding. The difference in baseline characteristics between groups before matching suggests that different phenotypes of patients received intensification with different study drugs. Even after successful matching for several key variables, it is impossible to eliminate such confounding by indication. Whether alternative approaches to address confounding (e.g., IPTW) would have produced different results is presently unclear. In addition, the quality of the data recorded under routine clinical care, often in non‐standardised ways, is typically lower than that recorded in the trial setting. Missingness for some key laboratory data (e.g., eGFR, UACR, triglycerides) was substantial: although imputation was used to enable matching, this likely increased the uncertainty around some secondary outcomes. The limited sample size reduced precision for some relevant secondary endpoints (including markers of kidney function and damage) and limited the ability to conduct clinically meaningful subgroup analyses. In addition, many patients were progressively lost to observation, such that the estimates at later timepoints were less reliable. Therefore, there is the need for larger and longer real‐world studies to comprehensively compare these important endpoints. The lack of information on adverse events, gastrointestinal tolerability, and medication adherence precluded a more proper evaluation of the persistent on treatment and on the factors associated with greater discontinuation of oral semaglutide. Even if the observation was longer than in the PIONEER‐2 trial, only a fraction of patients reached the 18‐month observation, and the duration of follow‐up was relatively short compared to chronic disease management. Finally, the study was conducted under specialised diabetes clinics in Italy, thereby limiting generalisability to primary care or other geographical settings.

## 5 CONCLUSION

This real‐world emulation of the PIONEER‐2 trial confirms the superior glycaemic efficacy of oral semaglutide versus empagliflozin in patients with T2D under routine care, with comparable weight loss, though with a lower persistence on treatment. These findings suggest that the advantages of oral semaglutide observed in trials can translate into practice.

## AUTHOR CONTRIBUTIONS

GPF designed the study, analysed data, and wrote the manuscript. EL, SP researched and analysed data, and wrote the manuscript. AG, MG, MS, MT, GP collected data and revised the manuscript. AA, AS, AC designed the study, provided support and supervision, and revised the manuscript. All authors approved the final version of the manuscript.

## FUNDING INFORMATION

This study was supported by the Italian Diabetes Society.

## CONFLICT OF INTEREST STATEMENT

GPF received honoraria, lecture, or advisory board fees from AstraZeneca, Boehringer, Guidotti, Lilly, Novartis, Novo Nordisk, and Sanofi. AG has received lecture fees from AstraZeneca, Lilly, and Novo Nordisk. GP reports consulting, advisory board, or speaking honoraria from Eli Lilly, consulting or participation on advisory boards for Bayer, and consulting for Novo Nordisk. AA received research grants, lecture, or advisory board fees from Merck Sharp & Dohme, AstraZeneca, Novartis, Boehringer‐Ingelheim, Sanofi, Mediolanum, Janssen, Novo Nordisk, Lilly, Servier, and Takeda. AS served on the advisory board of Novo Nordisk, Sankyo, and Sanofi and received speaker fees from AstraZeneca, Bayer, Lilly, Novo Nordisk, and Sanofi. AC received grants from AstraZeneca, Lilly, and Novo Nordisk. He also received speaker fees and provided advisory board services for Abbott, AstraZeneca, Boehringer Ingelheim Pharmaceuticals, Lilly, Merck Sharp & Dohme, Menarini, Novo Nordisk, Sanofi, Sigma‐Tau, and Takeda. EL, SP, MG, MS, and MT have nothing to disclose.

## PEER REVIEW

The peer review history for this article is available at https://www.webofscience.com/api/gateway/wos/peer-review/10.1111/dom.70151.

## Supporting information


Data S1.

## Data Availability

Restrictions apply to the availability of source data used in this study, mainly due to privacy regulation. Aggregate data may be available from the corresponding author at a reasonable request.
