# Supplementary material for: Effectiveness of oral semaglutide versus empagliflozin for the management of type 2 diabetes. PIONEER‐2 trial emulation with real‐world data
Source: Diabetes Obes Metab. 2025 Sep 24;27(12):7431–40. doi: 10.1111/dom.70151 (PMC12587239; doi:10.1111/dom.70151)
Supplement: Supplementary file 1 — Data S1. [file DOM-27-7431-s001.docx]

**Appendix**

**GLIMPLES Study investigators**

ABRUZZO. Maria Antonia Pompea Baldassarre, Gloria Formoso, Agostino Consoli (Endocrinology, Diabetes and Metabolism, University Chieti-Pescara).

LAZIO Gaetano Leto, Frida Leonetti (Diabetology Unit Latina, Department of Medical-Surgical Sciences and Biotechnologies, Sapienza University, Rome, Italy).

LOMBARDIA. Stefano Fazion (SSD di Diabetologia e Malattie Metaboliche, ASST-Mantova. Ospedale C. Poma), Giancarla Meregalli (Malattie Endocrine Centro Regionale Diabete Mellito ASST Bergamo Ovest, Treviglio, Italy).

PIEMONTE. Marco Zavattaro, Gianluca Aimaretti (Division of Endocrinology, Maggiore della Carità Hospital, Department of Translational Medicine, University of Piemonte Orientale, Novara). Elena Melchionda, Cristina Barale (Centro SS Diabetologia e Malattie metaboliche dell'AOU San Luigi Gonzaga di Orbassano).

SARDEGNA. Rosella Cau, Mariangela Ghiani (Unità di diabetologia Azienda Sanitaria Locale 8 Cagliari Quartu S. Elena).

SICILIA. Andrea Muscarà, Giuseppina Russo (Dipartimento di Medicina Clinica e Sperimentale, UOC Medicina Interna, Policlinico Universitario “G. Martino”, Messina). TOSCANA. Roberto Anichini (Diabetes Unit Area Pistoiese USL Toscana Centro).

TRENTINO-ALTO ADIGE. Bruno Fattor (Diabetology Service, Bolzano Hospital).

VENETO. Gian Paolo Fadini, Angelo Avogaro (Department of Medicine, University of Padova). Laura Nollino, Agostino Paccagnella (Diabetology Units, Conegliano and Treviso). Marco Strazzabosco (Endocrine, Metabolic and Nutrition Disease, Vicenza Hospital).

**DARWIN-Renal study Investigators**

ABRUZZO. Mariella Baldassarre, Agostino Consoli (Endocrinology, Diabetes and Metabolism, University Chieti-Pescara). Sara Morganet, Antonella Zugaro, Marco Giorgio Baroni (Diabetes and Andrology Unit, San Salvatore Hospital, University of L'Aquila).

CALABRIA. Francesco Andreozzi (Department of Medical and Surgical Sciences, University Magna Graecia of Catanzaro, Catanzar).

CAMPANIA. Adriano Gatti (Diabetology Service, ASL Napoli1). Stefano De Riu (Diabetology Unit ASL Napol1 Centro). Andrea Del Buono (Diabetology Unit, ASL Caserta).

EMILIA ROMAGNA. Raffaella Aldigeri, Riccardo Bonadonna (Division of Endocrinology and Metabolic Diseases, Azienda Ospedaliera-Universitaria di Parma,, Department of Medicine and Surgery, University of Parma), Alessandra Dei Cas (Division of Nutritional and Metabolic Sciences, Azienda Ospedaliero-Universitaria di Parma, University of Parma), Angela Vazzana, Monica Antonini, Valentina Moretti (Division of Endocrinology and Metabolic Diseases, Azienda Ospedaliera-Universitaria di Parma).

FRIULI VENEZIA GIULIA. Patrizia Li Volsi (Section of Endocrinology and Metabolism, Azienda Sanitaria Friuli Occidentale). Miranda Cesare, Giorgio Zanette (Section of Endocrinology and Metabolism, Pordenone Hospital, Azienda Sanitaria Friuli Orientale).

LAZIO. Silvia Carletti, Paola D'Angelo (Diabetology Unit, Sandro Pertini Hospital, ASL Roma2). Gaetano Leto, Frida Leonetti (Diabetology Unit Latina, Department of Medical-Surgical Sciences and Biotechnologies, Sapienza University, Rome, Italy). Luca D'Onofrio, Ernesto Maddaloni, Raffaella Buzzetti (Diabetology Unit, University of Rome "La Sapienza"). Fabiana Picconi, Simona Frontoni (Unit of Endocrinology, Diabetes and Metabolism, S. Giovanni Calibita Fatebenefratelli Hospital, Department of Systems Medicine, University of Rome Tor Vergata, Rome, Italy). Gisella Cavallo (Diabetology Unit, Department of Experimental Medicine, Sapienza University, Rome, Italy). Susanna Morano, Tiziana Filardi (University of Rome "La Sapienza", Dept. of Experimental Medicine, Unit of Diabetes and Complications, Clinica Medica V, Azienda Policlinico Umberto I, Roma). Francesca Cinti, Andrea Giaccari (Endocrinology and Diabetology, IRCCS Agostino Gemelli University Hospital Foundation, Department of Translational Medicine and Surgery, Catholic University of the Sacred Heart, Rome).

LOMBARDIA. Antonio C. Bossi (Unit of Diabetology, Humanitas Gavazzeni Institute, Bergamo). Giancarla Meregalli (Unit of Diabetology and Metabolic Diseases, Azienda Socio-Sanitaria Territoriale Bergamo Ovest, Treviglio, Bergamo). Fabrizio Querci (Diabetology Unit, Alzano Lombardo Hospital). Alessia Gaglio, Veronica Resi, Emanuela Orsi (Diabetes Unit, Foundation IRCCS Cà Granda Ospedale Maggiore Policlinico, Milan). Stefano Fazion (Diabetology and Metabolic Disease, ASST Mantova). Ivano G. Franzetti (Endocrinology and Diabetology Unit ASST Valle Olona). Cesare Berra (Diabetology and Endocrinology Unit, IRCCS Multimedica, Milan).

MARCHE. Silvia Manfrini (Diabetology Unit, Senigallia). Gabriella Garrapa, Giulio Lucarelli, Lara Riccialdelli (Diabetology Unit, Fano). Elena Tortato (Diabetology and Metabolic Disease, INRCA Ancona).

PIEMONTE. Marco Zavattaro, Gianluca Aimaretti (Division of Endocrinology, Maggiore della Carità Hospital, Department of Translational Medicine, University of Piemonte Orientale, Novara). Franco Cavalot (Diabetes and Metabolic Diseases Unit, San Luigi Gonzaga University Hospital, Turin). Guglielmo Beccuti, Fabio Broglio (Unit of Diabetology and Metabolism, Department of Medical Sciences, University of Turin, 10123 Torino, Italy.).

TRENTINO-ALTO ADIGE. Bruno Fattor (Diabetology Service, Bolzano Hospital).

PUGLIA. Giuliana Cazzetta (Diabetology Unit Tricase (Lecce)). Olga Lamacchia (Department of Medical and Surgical Sciences, University of Foggia). Anna Rauseo, Salvatore De Cosmo (Fondazione IRCCS Casa Sollievo della Sofferenza, San Giovanni Rotondo, Italy).

SARDEGNA. Rosella Cau, Mariangela Ghiani (UO diabetologia Quartu SE, ASL 8 Cagliari).

SICILIA. Antonino Di Benedetto (Diabetology Unit, University Hospital G. Martino, Messina). Antonino Di Pino, Salvatore Piro, Francesco Purrello (Internal Medicine, Garibaldi Nesima Hospital, Department of Clinical and Experimental Medicine, University of Catania). Lucia Frittitta, Agostino Milluzzo (Center for Diabetes and Obesity, Garibaldi Nesima Hospital, Department of Clinical and Experimental Medicine, University of Catania). Giuseppina Russo (Metabolic Disease and Internal Medicine, University Hospital of Messina).

TOSCANA. Anna Solini (Department of Surgical, Medical, Molecular and Critical Area Pathology, University of Pisa). Monia Garofolo, Giuseppe Penno, Stefano Del Prato (Department of Clinical and Experimental Medicine, University of Pisa, Pisa). Roberto Anichini (Diabetes Unit, Area Pistoiese USL Toscana Centro).

VENETO. Gian Paolo Fadini, Angelo Avogaro (Department of Medicine, University of Padova). Lucia Gottardo (Unit of Hypertension and Endocrine-Metabolic-Angiologic Disease, AULSS3 Venice). Mauro Rigato, Agostino Paccagnella (Diabetology Units, Conegliano and Treviso). Marco Strazzabosco (Endocrine, Metabolic and Nutrition Disease, Vicenza Hospital). Massimo Cigolini, Enzo Bonora (Division of Endocrinology, Diabetes and Metabolic Diseases, Department of Medicine, University of Verona).

**Table S1. Baseline clinical characteristics of participants before matching**.

|  | **Missing (%)** | **Oral semaglutide** | **Empagliflozin** | **SMD** |
| --- | --- | --- | --- | --- |
| **Demographics** |  | n=109 | n=3757 |  |
| Male sex, % | 0,0 | 70 (64.2) | 2516 (67.0) | 0.06 |
| Age, years | 0,0 | 65.0 (8.7) | 61.6 (8.8) | 0.39 |
| Duration, years | 1.4 | 10.5 (8.4) | 9.4 (7.7) | 0.12 |
| **Risk factors and lab results** |  |  |  |  |
| Body weight, kg | 10.3 | 81.8 (16.2) | 86.4 (16.4) | 0.21 |
| Body mass index, kg/m^2^ | 11.9 | 28.8 (4.9) | 30.7 (5.2) | 0.31 |
| Systolic blood pressure, mm Hg | 31.0 | 142.5 (16.4) | 137.5 (15.4) | 0.39 |
| Diastolic blood pressure, mm Hg | 31.1 | 80.4 (9.1) | 79.6 (8.3) | 0.08 |
| Fasting plasma glucose, mg/dl | 16.2 | 155.2 (43.7) | 167.9 (49.6) | 0.21 |
| HbA1c, % | 4.6 | 7.6 (1.1) | 8.2 (1.3) | 0.56 |
| Total cholesterol, mg/dl | 28.9 | 164.6 (31.8) | 169.3 (35.5) | 0.11 |
| HDL cholesterol, mg/dl | 31.6 | 48.8 (11.5) | 45.4 (9.9) | 0.21 |
| LDL cholesterol, mg/dl | 35.3 | 87.6 (26.7) | 91.7 (28.1) | 0.14 |
| Triglycerides, mg/dl | 29.9 | 138.9 (75.3) | 155.3 (93.0) | 0.16 |
| eGFR, ml/min/1.73 m^2^ | 27.7 | 86.0 (16.4) | 86.7 (13.6) | 0.12 |
| **Complications** |  |  |  |  |
| eGFR <60 ml/min/1.73 m^2^ | 27.7 | 10 (9.2) | 183 (4.9) | 0.22 |
| UACR >30 mg/g | 52.1 | 15 (13.8) | 552 (14.7) | 0.25 |
| Retinopathy, % | 22.1 | 14 (12.8) | 416 (11.1) | 0.07 |
| Cardiovascular disease, % | 28.0 | 11 (10.1) | 834 (22.2) | 0.54 |
| **Medications** |  |  |  |  |
| Metformin, % | 0.0 | 102 (93.6) | 3496 (93.1) | 0.02 |
| Sulphonylurea, % | 0.0 | 11 (10.1) | 248 (6.6) | 0.12 |
| Pioglitazone, % | 0.0 | 4 (3.7) | 66 (1.8) | 0.10 |
| Basal insulin, % | 0.0 | 7 (6.4) | 856 (22.8) | 0.67 |
| Statin, % | 13.3 | 82 (75.2) | 2739 (72.9) | 0.12 |
| Anti-platelet agents, % | 13.3 | 32 (29.4) | 2169 (57.7) | 0.36 |
| RAS blockers, % | 13.3 | 71 (65.1) | 2726 (72.6) | 0.13 |
| Beta-blockers, % | 13.3 | 26 (23.9) | 1216 (32.4) | 0.22 |
| Calcium channel blockers, % | 13.3 | 22 (20.2) | 726 (19.3) | 0.01 |
| Diuretics, % | 13.3 | 18 (16.5) | 999 (26.6) | 0.31 |

*Note*: Data are presented as mean (standard deviation) for continuous variables or as number (percentage) for categorical variables. The standardized mean difference (SMD) is shown for the between-group comparison before matching. The percentage of missing data that needed to be imputed for matching is also reported.

*Abbreviations*: eGFR, estimated glomerular filtration rate. UACR, urinary albumin/creatinine excretion ratio. RAS, renin angiotensin system.

**Table S2. Additional endpoints**. Data are presented as estimated mean difference (oral semaglutide – empagliflozin) and standard error from the mixed model for repeated measures. Negative values indicate a reduction in the oral semaglutide group. None of the reported difference was statistically significant.

| **Endpoint** | **Treatment policy estimand** | **Trial product estimand** |
| --- | --- | --- |
| Triglycerides, mg/dl | -0.7 (4.0) | -0.8 (4.1) |
| UACR, mg/g | -3.0 (5.5) | -2.9 (5.5) |
| eGFR, ml/min/1.73 m^2^ | -0.5 (0.8) | -0.5 (0.8) |

**Figure S1. Study flowchart**.

**Figure S2. Common support and covariate balance**. A) Plot showing the overlap in propensity scores (PS), known as common support. The bold lines indicate proportion of patients in each bin of PS and the dashed lines indicate the fitted distribution. B) Absolute standardized mean difference for each variable of interest before and after applying propensity score matching.

**Figure S3. Persistence on treatment**.
